# The Influence of Hormonal Factors on the Risk of Developing Cervical Cancer and Pre-Cancer: Results from the EPIC Cohort

**DOI:** 10.1371/journal.pone.0147029

**Published:** 2016-01-25

**Authors:** Esther Roura, Noémie Travier, Tim Waterboer, Silvia de Sanjosé, F. Xavier Bosch, Michael Pawlita, Valeria Pala, Elisabete Weiderpass, Núria Margall, Joakim Dillner, Inger T. Gram, Anne Tjønneland, Christian Munk, Domenico Palli, Kay-Tee Khaw, Kim Overvad, Françoise Clavel-Chapelon, Sylvie Mesrine, Agnès Fournier, Renée T. Fortner, Jennifer Ose, Annika Steffen, Antonia Trichopoulou, Pagona Lagiou, Philippos Orfanos, Giovanna Masala, Rosario Tumino, Carlotta Sacerdote, Silvia Polidoro, Amalia Mattiello, Eiliv Lund, Petra H. Peeters, H. B(as). Bueno-de-Mesquita, J. Ramón Quirós, María-José Sánchez, Carmen Navarro, Aurelio Barricarte, Nerea Larrañaga, Johanna Ekström, David Lindquist, Annika Idahl, Ruth C. Travis, Melissa A. Merritt, Marc J. Gunter, Sabina Rinaldi, Massimo Tommasino, Silvia Franceschi, Elio Riboli, Xavier Castellsagué

**Affiliations:** 1 Unit of Infections and Cancer, Cancer Epidemiology Research Program, Catalan Institute of Oncology (ICO)-IDIBELL, L’Hospitalet de Llobregat, Barcelona, Catalonia, Spain; 2 CIBER de Epidemiología y Salud Pública (CIBERESP), Madrid, Spain; 3 Unit of Nutrition, Environment and Cancer, Cancer Epidemiology Research Program, Catalan Institute of Oncology (ICO)-IDIBELL, L’Hospitalet de Llobregat, Barcelona, Catalonia, Spain; 4 Division of Molecular Diagnostics of Oncogenic Infections (F020), Research Program Infection, Inflammation and Cancer, German Cancer Research Center (DKFZ), Heidelberg, Germany; 5 Epidemiology and Prevention Unit, Fondazione IRCCS Istituto Nazionale dei Tumori, Milan, Italy; 6 Department of Medical Epidemiology and Biostatistics, Karolinska Institutet, Stockholm, Sweden; 7 Department of Community Medicine, Faculty of Health Sciences, UiT The Arctic University of Norway, Tromsø, Norway; 8 Department of Research, Cancer Registry of Norway, Oslo, Norway; 9 Genetic Epidemiology Group, Folkhälsan Research Center, Helsinki, Finland; 10 Microbiology Department, Universitat Autònoma de Barcelona, Hospital de la Santa Creu i Sant Pau, Barcelona, Catalonia, Spain; 11 Department of Laboratory Medicine, Karolinska Institutet, Stockholm, Sweden; 12 Diet, Genes and Environment, Danish Cancer Society Research Center, Copenhagen, Denmark; 13 Virus, Lifestyle and Genes, Danish Cancer Society Research Center, Copenhagen, Denmark; 14 Molecular and Nutritional Epidemiology Unit, Cancer Research and Prevention Institute–ISPO, Florence, Italy; 15 School of Clinical Medicine, University of Cambridge, Cambridge, United Kingdom; 16 Section for Epidemiology, Department of Public Health, Aarhus University, Aarhus, Denmark; 17 Inserm, Centre for research in Epidemiology and Population Health (CESP), U318, Nutrition, Hormones and Women’s Health team, F-94705, Villejuif, France; 18 Université Paris Sud, UMRS 318, F-94705, Villejuif, France; 19 Institut Gustave Roussy, F-94705, Villejuif, France; 20 Division of Cancer Epidemiology, German Cancer Research Center (DKFZ), Heidelberg, Germany; 21 Department of Epidemiology, German Institute of Human Nutrition Potsdam-Rehbruecke, Nuthetal, Germany; 22 Hellenic Health Foundation, Athens, Greece; 23 Department of Hygiene, Epidemiology and Medical Statistics, University of Athens Medical School, Athens, Greece; 24 Department of Epidemiology, Harvard School of Public Health, Boston, United States of America; 25 Cancer Registry and Histopathology Unit, "Civic—M.P. Arezzo" Hospital, Ragusa, Italy; 26 Unit of Cancer Epidemiology, University of Turin, Turin, Italy; 27 Centre for Cancer Epidemiology and Prevention (CPO Piemonte), Turin, Italy; 28 Human Genetics Foundation (HuGeF), Turin, Italy; 29 Dipartimento Di Medicina Clinica E Chirurgia, Federico II University, Naples, Italy; 30 MRC-PHE Centre for Environment and Health, Department of Epidemiology and Biostatistics, School of Public Health, Imperial College, London, United Kingdom; 31 Department of Epidemiology, Julius Center for Health Sciences and Primary Care, University Medical Center Utrecht, Utrecht, The Netherlands; 32 Department for Determinants of Chronic Diseases (DCD), National Institute for Public Health and the Environment (RIVM), Bilthoven, The Netherlands; 33 Department of Gastroenterology and Hepatology, University Medical Centre, Utrecht, The Netherlands; 34 Department of Epidemiology and Biostatistics, The School of Public Health, Imperial College London, London, United Kingdom; 35 Department of Social & Preventive Medicine, Faculty of Medicine, University of Malaya, Kuala Lumpur, Malaysia; 36 Public Health Directorate, Asturias, Spain; 37 Escuela Andaluza de Salud Pública, Instituto de Investigación Biosanitaria ibs.GRANADA, Hospitales Universitarios de Granada/Universidad de Granada, Granada, Spain; 38 Department of Epidemiology, Murcia Regional Health Council, IMIB-Arrixaca, Murcia, Spain; 39 Department of Health and Social Sciences, Universidad de Murcia, Murcia, Spain; 40 Navarra Public Health Institute, Pamplona, Spain; 41 Navarra Institute for Health Research (IdiSNA), Pamplona, Spain; 42 Public Health Division of Gipuzkoa, BIODonostia Research Institute, Basque Health Department, Bilbao, Spain; 43 BBMRI.se Service Center for Southern Sweden, Lund University, Medicon Village, Lund, Sweden; 44 Department of Radiation Sciences, Umeå University, Umeå, Sweden; 45 Department of Clinical Sciences, Obstetrics and Gynecology, Umeå University, Umeå, Sweden; 46 Cancer Epidemiology Unit, Nuffield Department of Population Health, University of Oxford, Oxford, United Kingdom; 47 International Agency for Research on Cancer, Lyon, France; Albert Einstein College of Medicine, UNITED STATES

## Abstract

**Background:**

In addition to HPV, high parity and hormonal contraceptives have been associated with cervical cancer (CC). However, most of the evidence comes from retrospective case-control studies. The aim of this study is to prospectively evaluate associations between hormonal factors and risk of developing cervical intraepithelial neoplasia grade 3 (CIN3)/carcinoma in situ (CIS) and invasive cervical cancer (ICC).

**Methods and Findings:**

We followed a cohort of 308,036 women recruited in the European Prospective Investigation into Cancer and Nutrition (EPIC) Study. At enrollment, participants completed a questionnaire and provided serum. After a 9-year median follow-up, 261 ICC and 804 CIN3/CIS cases were reported. In a nested case-control study, the sera from 609 cases and 1,218 matched controls were tested for L1 antibodies against HPV types 11,16,18,31,33,35,45,52,58, and antibodies against *Chlamydia trachomatis* and *Human herpesvirus 2*. Multivariate analyses were performed to estimate hazard ratios (HR), odds ratios (OR) and corresponding 95% confidence intervals (CI). The cohort analysis showed that number of full-term pregnancies was positively associated with CIN3/CIS risk (p-trend = 0.03). Duration of oral contraceptives use was associated with a significantly increased risk of both CIN3/CIS and ICC (HR = 1.6 and HR = 1.8 respectively for ≥15 years *versus* never use). Ever use of menopausal hormone therapy was associated with a reduced risk of ICC (HR = 0.5, 95%CI: 0.4–0.8). A non-significant reduced risk of ICC with ever use of intrauterine devices (IUD) was found in the nested case-control analysis (OR = 0.6). Analyses restricted to all cases and HPV seropositive controls yielded similar results, revealing a significant inverse association with IUD for combined CIN3/CIS and ICC (OR = 0.7).

**Conclusions:**

Even though HPV is the necessary cause of CC, our results suggest that several hormonal factors are risk factors for cervical carcinogenesis. Adherence to current cervical cancer screening guidelines should minimize the increased risk of CC associated with these hormonal risk factors.

## Introduction

Cervical cancer (CC) is the fourth most common cancer among women worldwide with an estimated 528,000 new cases and the fourth most common cause of female death from cancer with an estimated 266,000 deaths in 2012 [1]. Human papillomavirus (HPV) is one of the most common sexually transmitted infections worldwide. In fact, most sexually active women will be infected with HPV during their lifetime, although the majority of HPV infections are cleared within 2 years [2,3]. HPV genotypes are classified as low-risk or high-risk based on their association with cervical cancer (CC) [4]. It is well established that persistent infection with high-risk HPV genotypes is the necessary although not sufficient cause of CC [5]. Thus, the involvement of other factors, in addition to HPV, is needed to induce cervical carcinogenesis. High parity and hormonal contraceptives have long been recognized as potential cofactors of CC [5]. A comprehensive review conducted by the International Agency for Research on Cancer (IARC) classified the use of combined oral contraceptives (OC) as carcinogenic to humans, and this was partly based on the reported associations with CC [6]. A collaborative pooled reanalysis evaluating CC, hormonal contraceptives and parity found an increased risk of CC in current and long-term OC users, a reduced risk after stopping these hormones [7], and positive associations with both number of full-term pregnancies (FTP) and an early age at first FTP [8]. In addition, results from the European Prospective Investigation into Cancer and Nutrition (EPIC) showed that circulating levels of sex steroid hormones testosterone and possibly estradiol were also positively involved in the etiology of CC [9]. However, even though these associations are generally consistent across studies, it must be noted that the evidence for a role of hormones in cervical carcinogenesis is mostly derived from retrospective case-control studies that did not always take into account HPV. Thus the aim of this study is to prospectively examine potential associations between hormonal factors and risk of developing cervical cancer and pre-cancer using data from a large prospective study that additionally uses serological markers of HPV exposure.

## Materials and Methods

### The EPIC cohort study

The EPIC study is a large prospective cohort study including 521,448 participants (367,993 women and 153,455 men) recruited between 1992 and 2000 through 23 centres in 10 European countries: Denmark, France, Germany, Greece, Italy, the Netherlands, Norway, Spain, Sweden, and the United Kingdom. Most of the EPIC participants were between the ages of 35 and 70 years. The study procedures have been described in detail elsewhere [9,10]. At recruitment, participants gave their written informed consent and completed questionnaires on their diet, medical and lifestyle history. They were also invited to provide blood samples for future testing of markers of interest. The EPIC study was approved by the ethical review committees from each center.

### Study population

Of the approximately 370,000 women enrolled in the study, women were not eligible for this analysis if they had prevalent cancer or pre-cancer (n = 22,180), incomplete follow-up (n = 2,295), hysterectomy (n = 34,973) or incomplete lifestyle questionnaire (n = 509) at baseline. This left a total of 308,036 women in these analyses.

### Identification of cases and follow-up

Cases of cervical intraepithelial neoplasia grade 3 (CIN3)/carcinoma in situ (CIS) and invasive cervical cancer (ICC) were identified through several methods, including a record linkage with population-based cancer registries (Denmark, Italy, the Netherlands, Norway, Spain, Sweden and the United Kingdom), health insurance records, hospital-based cancer and pathology registries and active follow-up of subjects (France, Germany and Greece). Data on vital status were obtained from mortality registries at regional and national level. Cervical cancer cases included only those women with first primary incident cancer according to the International Classification of Diseases, 10th revision (code C53: cervix uteri). Contrary to ICC, ascertainment of CIN3/CIS cases was not systematically done in all cancer registries and EPIC centers. Follow-up time was calculated between the date at recruitment and the date at diagnosis for cases or the date at censoring (death, loss of follow-up or end of follow-up) for non-cases. The end of follow-up ranged from December 2003 to December 2006, depending on the center. The median follow-up time in this cohort was around 9 years (25^th^-75^th^ percentile: 7.5–10.8 years) contributing a total of 2,775,235 person-year. Among the 308,036 women included in the final analysis 1,065 cases were identified: 261 ICC cases and 804 CIN3/CIS cases. Detailed tumor histology was specified for 953 cases (89%), of which 901 (95%) were classified as squamous cell carcinoma (SCC, 712 in situ and 189 invasive) and 52 (5%) as adenocarcinoma (9 in situ and 43 invasive).

### Nested case-control study

A nested case-control study within the EPIC cohort was conducted to allow for the adjustment by serological markers of HPV and other sexually transmitted infections (STIs), even though this strategy involves a lower number of subjects. For each case with available blood sample, two matched control subjects were randomly selected from the cancer-free cohort of women that were at risk at the time of diagnosis of the corresponding case. Matching criteria included: study center, age at recruitment (5 year intervals), menopausal status (pre-, peri- and postmenopausal), follow-up time, date, time and fasting status at blood collection, and, among premenopausal women, phase of the menstrual cycle [9]. Approximately 70% of ICC cases and 53% of CIN3/CIS cases identified in the cohort provided serum samples, yielding a total of 609 cases (184 ICC and 425 CIN3/CIS) and 1,218 controls for the analyses.

### Serological testing

HPV serology was performed at the German Cancer Research Centre in Heidelberg, Germany. Assay procedures have been explained in detail previously [11,12]. Briefly, antibodies to the capsid protein L1 of high-risk mucosal HPV types 16, 18, 31, 33, 35, 45, 52, and 58, and of low-risk mucosal HPV type 11 were tested by Glutathione S-transferase capture and fluorescent bead-based multiplex serology [13–15]. For the nested case-control analyses, HPV L1 seropositivity refers to that for at least one of the nine HPV types analyzed, and high-risk HPV L1 seropositivity refers to positivity for at least one of the eight high-risk HPV types analyzed.

Serum antibodies against *Chlamydia trachomatis* (CT) and *Human herpesvirus 2* (HHV-2) were tested at the Hospital of Santa Creu i Sant Pau in Barcelona, Spain. We used the commercial assay Chlamydia MIF IgG (Focus Diagnostics, Cypress, CA, USA) for the detection of CT IgG serum antibodies performed by microimmunofluorescence, and the commercial kit HerpeSelect® 2 ELISA IgG (Focus Diagnostics, Cypress, CA, USA) for the detection of antibodies against HHV-2 evaluated by an enzyme immunoassay. The positive results of HHV-2 antibodies were confirmed by a membrane-based immunoassay with the **biokit**HSV-2 Rapid Test (Biokit USA, Lexington, MA, USA).

All serological assays were performed blinded to the subject’s characteristics.

### Statistical analyses

Multivariate hazard ratios (HR) and 95% confidence intervals (CI) were estimated to evaluate the risk of CIN3/CIS and ICC in relation to several hormonal risk factors using Cox proportional hazard regression models. In all analyses, age was used as the underlying time variable, and the models were stratified by age at recruitment (in one-year categories) and study center. The proportional hazards assumption was evaluated for all models fitted using tests based on weighted residuals. Tests for linear trend were performed using continuous variables.

We estimated HR for variables related with hormonal factors collected at recruitment. The parity-related variables evaluated in these analyses were the following: ever FTP (never, ever), number of FTP (1, 2, 3, ≥4), age at first FTP (≥30, 25–29, 21–24, ≤20 years old), and number of induced abortions (1, ≥2) if any (never, ever). Self-reported baseline menopausal status was defined as postmenopausal (no menses in the last 12 months or bilateral ovariectomy), perimenopausal (<9 menses in the past 12 months), and premenopausal (regular menses in the past 12 months). Women with unknown menopausal status were classified as postmenopausal if they were 55 years old or more, perimenopausal if they were between 46 and 55 years of age and premenopausal when they were less than 46 years of age at recruitment. We estimated the cumulative years of menstrual cycling as the difference between the age at menopause (for postmenopausal women) or of the age at recruitment (for pre- and perimenopausal women) and the age at menarche minus the total time being pregnant (number of FTP x 9 months) and the total time using OCs; this variable was categorized in quintiles: ≤19.50, 19.51–26.76, 26.77–31.50, 31.51–35.50, ≥35.51 years. The exogenous hormone-related factors evaluated included: OC use (never, ever, past, current), duration of OC use (≤1, 2–4, 5–9, 10–14, ≥15 years), latency of OC use or time since first use (≤10, 11–20, ≥21 years), and recency of OC use or time since last use (≤5, 6–14, ≥15 years) among past OC users. Use of intrauterine device (IUD) was also analyzed and dichotomized as never and ever IUD use. Among post- and perimenopausal women, use of menopausal hormone therapy (HT) was evaluated as: HT use (never, ever, past, current), duration of HT use (≤1, 2–4, ≥5 years) and HT formulation (estrogen alone, progesterone alone, combination of estrogen/progesterone). Ovariectomy was also assessed (no, unilateral, bilateral). Combined variables using number of FTP and age at first FTP, duration and recency of OC use, and number of FTP and duration of OC use were also created and evaluated. The following variables were also analyzed but not included in the tables presented because they were either collinear with the final selected variables or not associated with risk in any of the analyses: time since first FTP, pregnancies, live births, stillbirths, miscarriages, breastfeeding, age started and stopped OC use, use of different contraceptives (condoms, spermicidal creams, tubal ligation, rhythm methods, diaphragm, vasectomy), age started HT use, type of HT (oral, injectable, topical), menopausal status, age at menarche, and age at menopause.

We used stepwise regression modeling to assess potential confounding by other variables such as: body mass index (BMI, underweight (<18.5), normal (18.5–25), overweight (25–30), obese (≥30)), marital status (single, married/cohabiting, divorced/separated, widowed),education level (none, primary school, technical/professional school, secondary school, university degree), physical activity (inactive, moderately inactive, moderately active, and active, using a validated Cambridge Physical Activity Index [16]), and smoking habits (never, former and duration <15 years, former and duration ≥15 years, current and intensity <10 cig/day, current and intensity ≥10 cig/day). Number of FTP (0, 1, 2, 3, ≥4), OC use and duration (never, past for <10 years, past for ≥10 years, current for <10 years, current for ≥10 years), and menopausal status with HT use (premenopausal, peri- and postmenopausal and non HT users, peri- and postmenopausal and HT users) were used as adjusting variables when appropriate.

In the nested case-control analysis, multivariate odds ratios (OR) and 95% CI were estimated to evaluate the risk of CIN3/CIS and ICC in relation to hormonal factors using conditional logistic regression models. Models were adjusted for HPV L1 serology, CT serology, HHV-2 serology, and other potential confounding factors (BMI, marital status, education level, physical activity, smoking habits, OC use and duration, number of FTP and menopausal status with HT use (when appropriate)). Unconditional logistic regression analyses among all cases and HPV L1 seropositive controls were also performed, including the matching variables in the models as adjusting covariates. Tests for interaction among hormonal variables and other risk factors were based on the likelihood ratio test comparing the models with and without the interaction terms.

When applicable, variables included a missing or unknown category in order to avoid the exclusion of participants in the regression models. All statistical tests were 2-tailed, and p-values below 0.05 were considered statistically significant. Statistical analyses were performed using the R programing language (R Development Core Team, 2005, http://www.R-project.org).

### Ethics statement

All participants gave written informed consent, and the study was approved by the local ethics committees in the participating countries (Athens: University of Athens Medical School; Cambridge: Norwich District Ethics Committee; Denmark (Aarhus, Copenhagen): The National Committee on Health Research Ethics; France (Paris): Comité de Protection des Personnes; Heidelberg: Ethics Committee of the Heidelberg University Medical School; International Agency for Research on Cancer: IARC Ethics Committee; Imperial College: Imperial College Research Ethics Committee [ICREC]; Italy (Florence, Milan, Naples, Ragusa, Turin): Comitato Etico Indipendente, Fondazione IRCCS Istituto Nazionale dei Tumori, Milano; Florence: Comitato Etico Locale Azienda Sanitaria di Firenze; Malmo: Ethics Committee of Lundst University; Netherlands (Bilthoven and Utrecht): The Medical Ethical Committee (METC = Medisch Ethische Toetsingscommissie) of the University Medical Center Utrecht (UMCU), Utrecht, the Netherlands; Norway: Regional ethical committee for Northern Norway and the Norwegian Data Inspectorate; Oxford: Scotland A Research Ethics Committee; Potsdam: Ethikkommission der Landesärztekammer Brandenburg Cottbus, Deutschland; Spain (Asturias, Barcelona, Granada, Murcia, Navarre, San Sebastian): CEIC Comité de Ética de Investigación Clínica; Turin: Human Genetics Foundation Torino: Ethics Committee; Umea: Umea Regional Ethical Review Board) and the Internal Review Board of the International Agency for Research on Cancer.

## Results

Baseline characteristics for cases and non-cases included in the cohort analysis have been reported previously (S1 Table) [11]. In brief, most CIN3/CIS cases were recruited from the United Kingdom (37.1%), Sweden (20.8%) and Norway (15.0%), and most of the ICC cases were from Sweden (16.9%), the United Kingdom (13.4%), Denmark (12.6%) and France (11.5%). CIN3/CIS cases were younger than ICC cases. As compared to non-cases, women with CIN3/CIS or ICC were more likely to be single or separated, to smoke, to have ever used OCs, and to be premenopausal. The characteristics of the women included in the nested case-control analysis have already been described in two previous articles [11,12].

Table 1 shows associations between factors related to endogenous hormones and risk of developing CIN3/CIS and ICC by study design (full cohort study and nested case-control study). In both analyses the risk of CIN3/CIS increased significantly with increasing number of FTP. In contrast, for ICC, a non significant decreased risk was observed among women who ever had a FTP. A decreased risk of CIN3/CIS and ICC was observed with decreasing age at first FTP, but only in the case-control study and this was only statistically significant for CIN3/CIS. In both study designs, a non significant decreased risk of CIN3/CIS was observed among women who had more than one induced abortion. However, for ICC an increased risk was found among women who had at least one induced abortion but the association was statistically significant only in the cohort study. When analyses were adjusted or stratified by number of FTP, the magnitude of the point estimates of the associations with both outcomes remained mostly unchanged (data not shown). Women with higher lifetime years of menstrual cycles had a lower risk of CIN3/CIS in both studies and of ICC in the cohort study.

**Table 1:** Risk of CIN3/CIS and ICC of the cervix according to factors related with endogenous hormones.

Risk factor	Cohort study				Nested case-control study			
	CIN3/CIS		ICC		CIN3/CIS		ICC	
	Non-cases / Cases	HR (95% CI) ^1^	Non-cases / Cases	HR (95% CI) ^1^	Controls / Cases	OR (95% CI) ^2^	Controls / Cases	OR (95% CI) ^2^
**Number of FTP**								
Never	45,228 / 191	1.0 (ref)	45,419 / 45	1.0 (ref)	139 / 51	1.0 (ref)	43 / 30	1.0 (ref)
Ever	246,823 / 516	**1.5 (1.2–1.9)**	247,339 / 191	0.7 (0.5–1.0)	543 / 288	**2.0 (1.2–3.2)**	281 / 131	0.8 (0.4–1.5)
1	44,007 / 116	**1.4 (1.0–1.8)**	44,123 / 36	0.7 (0.5–1.2)	132 / 71	1.7 (1.0–2.9)	42 / 26	0.8 (0.3–2.0)
2	114,956 / 237	**1.4 (1.1–1.9)**	115,193 / 82	0.7 (0.4–1.0)	247 / 132	**2.1 (1.2–3.4)**	153 / 53	0.5 (0.2–1.1)
3	54,107 / 94	**1.4 (1.0–1.9)**	54,201 / 40	0.7 (0.4–1.2)	97 / 46	**2.1 (1.2–3.8)**	55 / 28	1.0 (0.4–2.3)
≥4	23,545 / 51	**2.3 (1.6–3.3)**	23,596 / 23	0.9 (0.5–1.6)	42 / 28	**2.6 (1.3–5.3)**	18 / 16	1.5 (0.5–4.4)
*p for trend among ever ftp*		***0*.*03***		*0*.*4*		*0*.*2*		***0*.*02***
**Age at first FTP (years)** ^3^								
≥30	35,122 / 71	1.0 (ref)	35,193 / 22	1.0 (ref)	69 / 39	1.0 (ref)	22 / 13	1.0 (ref)
25–29	85,567 / 161	1.1 (0.8–1.4)	85,728 / 66	1.2 (0.7–2.0)	164 / 90	1.0 (0.6–1.8)	104 / 48	0.8 (0.2–2.9)
21–24	89,750 / 181	1.1 (0.8–1.5)	89,931 / 65	1.0 (0.6–1.6)	192 / 106	0.7 (0.4–1.3)	107 / 41	0.6 (0.2–1.9)
≤20	35,372 / 101	1.2 (0.9–1.7)	35,473 / 36	1.1 (0.6–2.0)	113 / 52	0.5 (0.3–1.1)	47 / 27	1.0 (0.2–4.3)
*p for trend among ever ftp*		*0*.*2*		*0*.*8*		***0*.*03***		*0*.*9*
**Number of induced abortions** ^4^								
Never	157,566 / 278	1.0 (ref)	157,844 / 99	1.0 (ref)	295 / 166	1.0 (ref)	180 / 70	1.0 (ref)
Ever	41,079 / 77	0.9 (0.7–1.1)	41,156 / 53	**1.9 (1.3–2.7)**	84 / 45	0.6 (0.4–1.0)	57 / 41	1.7 (0.8–3.4)
1	28,058 / 63	1.0 (0.7–1.3)	28,121 / 36	**1.9 (1.3–2.8)**	63 / 36	0.6 (0.4–1.2)	38 / 26	1.4 (0.6–3.4)
≥2	12,722 / 13	0.6 (0.3–1.0)	12,735 / 17	**1.8 (1.0–3.2)**	21 / 9	0.5 (0.2–1.2)	18 / 15	2.1 (0.8–6.1)
**Cumulative years of menstrual cycles without OCs (quintiles)** ^5^								
Quintile 1	47,020 / 270	1.0 (ref)	47,290 / 57	1.0(ref)	126 / 78	1.0 (ref)	43 / 26	1.0 (ref)
Quintile 2	46,953 / 118	0.9 (0.7–1.1)	47,071 / 29	**0.5 (0.3–0.8)**	124 / 64	1.0 (0.6–1.7)	39 / 28	1.6 (0.6–3.8)
Quintile 3	48,557 / 91	0.8 (0.6–1.1)	48,648 / 49	0.8 (0.5–1.3)	106 / 57	0.8 (0.5–1.4)	66 / 22	0.5 (0.2–1.2)
Quintile 4	47,855 / 63	**0.7 (0.5–1.0)**	47,918 / 36	0.6 (0.4–1.1)	111 / 43	**0.5 (0.3–0.9)**	60 / 39	1.0 (0.4–2.3)
Quintile 5	44,733 / 47	0.7 (0.5–1.1)	44,780 / 24	**0.4 (0.2–0.8)**	114 / 49	**0.5 (0.3–0.9)**	74 / 23	0.7 (0.3–1.7)
*p for trend*		***0*.*009***		***0*.*005***		***0*.*01***		*0*.*6*

CIN3: cervical intraepithelial neoplasia grade 3; CIS: carcinoma in situ; ICC: invasive cervical cancer; HR: hazard ratio; OR: odds ratio; CI: confidence interval; FTP: full-term pregnancy; OC: oral contraceptives; HT: hormone therapy. The number of cases does not add up the total number of cases because of missing values. Bold font indicates a statistically significant effect (p<0.05).

^1^ Models were adjusted by body mass index, marital status, level education, physical activity, smoking habits, OC use and duration and menopausal status with HT use.

^2^ Conditional regression models were adjusted by HPV L1 serology, *Chlamydia trachomatis* serology, *Human herpesvirus 2* serology, body mass index, marital status, level education, physical activity, smoking habits, OC use and duration and menopausal status with HT use. See methods for list matching variables.

^3^ Among parous women.

^4^ Excludes Bilthoven, Sweden and Norway because information was not collected for this variable.

^5^ Model not adjusted by OC use and duration because of co-linearity with cumulative duration of menstrual cycles. For cohort study, quintiles correspond to: q1: ≤19.50; q2: 19.51–26.76; q3: 26.77–31.50; q4: 31.51–35.50; q5: ≥35.51. For nested case-control study, quintiles correspond to: q1: ≤14.66; q2: 14.67–24.23; q3: 24.24–29.17; q4: 29.18–33.75; q5: ≥33.76.

When analyses were restricted to parous women and mutually adjusted for number of FTP and age at first FTP, the effect of number of FTP for CIN3/CIS risk was maintained in the full cohort analysis (HR = 1.6, 95% CI: 1.1–2.3), and was of borderline statistical significance in the nested case-control study (OR = 2.1, 95% CI: 1.0–4.6; data not shown). No associations were found for ICC risk.

Table 2 summarizes associations with factors related to exogenous hormones. Current use of OCs and increasing years of use were both associated with CIN3/CIS and ICC in the cohort study, even though the trend was only statistically significant for CIN3/CIS. As compared to current users, increasing years since last OC use was associated with a reduction in the risk of developing CIN3/CIS in the cohort study. In the case-control study associations were found in the same direction but did not reach statistical significance. Ever use of HT among peri- and postmenopausal women significantly decreased the risk of ICC in both studies. We found a decreased risk of ICC with duration of HT use but the trend did not reach statistical significance. In contrast, for CIN3/CIS we found a statistically significant reduced risk with years of HT in the case-control study. Similar results were obtained when analyses where restricted to postmenopausal women only (data not shown). We also assessed the effect of HT formulation finding a non significant increased risk of CIN3/CIS for users of menopausal estrogens alone (HR = 1.7, 95% CI: 0.9–3.1, for ever *versus* never users) and a lack of association for combined formulations (data not shown). The effect of progestin alone could not be assessed due to the low number of exposed subjects. For ICC, most exposed cases used some kind of combination of hormones showing a borderline inverse association (HR = 0.6, 95% CI: 0.3–1.0, for ever *versus* never users). There were not enough cases to assess the effect of estrogen or progestin alone (data not shown). Concerning IUD use, a non significant inverse association with both CIN3/CIS and ICC risk was observed in the nested case-control study.

**Table 2:** Risk of CIN3/CIS and ICC of the cervix according to factors related with exogenous hormones.

Risk factor	Cohort study				Nested case-control study			
	CIN3/CIS		ICC		CIN3/CIS		ICC	
	Non-cases / Cases	HR (95% CI) ^1^	Non-cases / Cases	HR (95% CI) ^1^	Controls / Cases	OR (95% CI) ^2^	Controls / Cases	OR (95% CI) ^2^
**IUD use** ^3^								
Never	170,843 / 371	1.0 (ref)	171,214 / 144	1.0 (ref)	325 / 160	1.0 (ref)	194 / 106	1.0 (ref)
Ever	63,677 / 136	1.1 (0.9–1.4)	63,813 / 43	0.9 (0.6–1.3)	160 / 82	0.8 (0.5–1.3)	70 / 28	0.6 (0.3–1.2)
**OC use**								
Never	121,117 / 169	1.0 (ref)	121,286 / 76	1.0 (ref)	225 / 99	1.0 (ref)	139 / 56	1.0 (ref)
Ever	176,993 / 548	1.1 (0.9–1.3)	177,541 / 165	**1.6 (1.1–2.3)**	466 / 244	1.1 (0.8–1.5)	186 / 109	1.5 (0.8–2.6)
Past	152,658 / 411	1.0 (0.9–1.3)	153,069 / 134	**1.6 (1.1–2.2)**	392 / 197	1.0 (0.7–1.5)	158 / 86	1.3 (0.7–2.4)
Current	17,384 / 127	**1.8 (1.4–2.4)**	17,511 / 22	**2.2 (1.3–4.0)**	57 / 41	1.7 (0.9–3.1)	24 / 17	2.2 (0.7–6.7)
**Duration of OC use (years)**								
Never	121,117 / 169	1.0 (ref)	121,286 / 76	1.0 (ref)	225 / 99	1.0 (ref)	139 / 56	1.0 (ref)
≤1	31,867 / 78	1.0 (0.8–1.3)	31,945 / 27	1.5 (0.9–2.4)	180 / 82	1.0 (0.6–1.5)	76 / 36	1.1 (0.6–2.2)
2–4	40,168 / 127	1.1 (0.8–1.4)	40,295 / 27	1.3 (0.8–2.0)				
5–9	38,816 / 136	1.1 (0.9–1.4)	38,952 / 41	**2.0 (1.3–3.0)**	108 / 54	1.1 (0.7–1.7)	38 / 22	1.6 (0.7–3.7)
10–14	26,969 / 90	1.2 (0.9–1.6)	27,059 / 26	1.6 (1.0–2.6)	138 / 94	**1.6 (1.0–2.5)**	63 / 42	1.5 (0.7–3.1)
≥15	23,395 / 82	**1.6 (1.2–2.2)**	23,477 / 28	**1.8 (1.1–2.9)**				
*p for trend among OC users*		***0*.*01***		*0*.*2*		*0*.*2*		*0*.*9*
**Recency of OC use (years)** ^4^^**,**^^5^								
Current	7,678 / 95	1.0 (ref)	7,773 / 8	1.0 (ref)	32 / 20	1.0 (ref)	10 / 12	1.0 (ref) ^7^
≤5	9,662 / 79	**0.7 (0.5–1.0)**	9,741 / 11	1.1 (0.4–2.8)	30 / 21	1.0 (0.2–4.2)		
6–14	17,368 / 60	**0.6 (0.4–0.8)**	17,428 / 14	0.8 (0.3–2.0)	44 / 21	0.4 (0.1–1.9)	27 / 11	-
≥15	25,681 / 45	**0.6 (0.4–0.9)**	25,726 / 25	1.0 (0.4–2.5)	48 / 22	0.6 (0.1–5.0)	32 / 20	-
*p for trend among past OC users*		*0*.*2*		*0*.*7*		*-*		*-*
**HT use** ^6^								
Never	114,271 / 149	1.0 (ref)	114,420 / 94	1.0 (ref)	170 / 83	1.0 (ref)	107 / 67	1.0 (ref)
Ever	63,839 / 131	1.2 (0.9–1.5)	63,970 / 31	**0.5 (0.4–0.8)**	149 / 84	0.9 (0.6–1.5)	72 / 18	**0.3 (0.1–0.7)**
Past	18,508 / 22	1.0 (0.6–1.5)	18,530 / 11	0.6 (0.3–1.2)	42 / 17	0.7 (0.3–1.4)	19 / 8	0.6 (0.2–2.0)
Current	43,110 / 102	1.3 (1.0–1.7)	43,212 / 19	**0.5 (0.3–0.8)**	102 / 61	1.1 (0.7–1.9)	51 / 10	**0.2 (0.1–0.5)**
**Duration of HT use (years)** ^6^								
Never	114,271 / 149	1.0 (ref)	114,420 / 94	1.0 (ref)	170 / 83	1.0 (ref)	107 / 67	1.0 (ref)
≤1	22,819 / 43	1.3 (0.9–1.9)	22,862 / 12	0.7 (0.4–1.2)	42 / 28	1.2 (0.6–2.5)	39 / 11	**0.4 (0.1–1.0)**
2–4	19,032 / 32	1.0 (0.7–1.5)	19,064 / 9	0.6 (0.3–1.1)	44 / 15	0.5 (0.2–1.1)		
≥5	15,834 / 27	1.0 (0.7–1.6)	15,861 / 7	**0.4 (0.2–0.9)**	49 / 19	0.6 (0.3–1.4)	26 / 5	**0.1 (0.03–0.6)**
*p for trend among menopausal hormones users*		*0*.*2*		*0*.*4*		*-*		-

CIN 3: cervical intraepithelial neoplasia grade 3; CIS: carcinoma in situ; ICC: invasive cervical cancer; HR: hazard ratio; OR: odds ratio; CI: confidence interval; IUD: intrauterine device; OC: oral contraceptives; HT: hormone therapy. The number of cases does not add up the total number of cases because of missing values. Bold font indicates a statistically significant effect (p<0.05).

^1^ Models were adjusted by body mass index, marital status, level education, physical activity, smoking habits, number of full-term pregnancies and menopausal status with HT use.

^2^ Conditional regression models were adjusted by HPV L1 serology, *Chlamydia trachomatis* serology, *Human herpesvirus 2* serology, body mass index, marital status, level education, physical activity, smoking habits, number of full-term pregnancies and menopausal status with HT use. See methods for list matching variables.

^3^ Excludes Bilthoven, Sweden and Norway because information was not collected for this variable.

^4^ Among OC users (excluding non OC users).

^5^ Excludes Bilthoven, France, Germany, Sweden, Denmark and Norway because information was not collected for this variable.

^6^ Among peri and postmenopausal women (excluding premenopausal women) and models also adjusted by OC use and duration but not adjusted by menopausal status with HT use. ^7^ Risk estimates were not estimated due to lack of power in the model.

When the combined effect of duration and recency of OC use was evaluated, the risk of CIN3/CIS declined progressively with increasing years since last use (data not shown). This pattern was not observed for ICC risk.

In both study designs, the combined effect of number of FTP and duration of OC use was analyzed, and showed a significant increased risk of CIN3/CIS with increasing number of FTP within each category of OC use (S2 Table). Women with 4 or more FTP had a four-fold risk within each category of OC use as compared to women who were nulliparous and never used OCs. The test of interaction between OC use and FTP reached statistical significance in the cohort study (p = 0.004). In contrast, for ICC we only found a marginal increased risk among multiparous women who used OCs for more than 5 years in the cohort study.

Concerning associations by histological type, the risk of SCC showed the same overall pattern: increased risk of CIS with number of FTP and years of OC use and decreased risk with years of HT use, and increased risk of invasive SCC with years of OC use and decreased risk with years of HT use (data not shown). Regarding adenocarcinomas, associations could not be evaluated accurately because of the small number of cases (52 cases in the cohort study and 33 cases in the nested case-control study for both in situ and invasive adenocarcinomas). Nevertheless, these analyses showed non significant and weak positive associations with number of FTP and OC use for invasive adenocarcinoma, and a non significant inverse association with HT use (data not shown).

Analyses restricted to countries with screening programs (United Kingdom, Sweden, Denmark, Norway) did not substantially change the magnitude of the associations for the several risk factors (data not shown).

Table 3 shows associations restricted to all cases and HPV L1 seropositive controls in the nested case-control study. In this analysis number of FTP was strongly associated with an increased risk of CIN3/CIS. For induced abortions we found opposite effects for CIN3/CIS *versus* ICC (OR = 0.5 and OR = 1.7 respectively, for ever *versus* never). Current use and duration of OCs increased the risk of both CIN3/CIS and ICC although the associations were not statistically significant. HT use and duration was inversely and significantly associated with ICC. Finally, IUD use was inversely associated with both CIN3/CIS and ICC risk without statistical significance, although when we combined CIN3/CIS and ICC the significance emerged (OR = 0.7, 95% CI: 0.5–0.96; data not shown). Associations were broadly similar in analyses restricted to all cases and high-risk HPV seropositive controls and in analyses including only HPV seropositive cases and controls (data not shown), revealing a significant inverse association between IUD use and ICC among HPV seropositive women (OR = 0.3, 95% CI: 0.1–0.9; data not shown).

**Table 3:** Multivariate odds ratios for the association between factors related to endogenous and exogenous hormones and CIN3/CIS and ICC cases among all cases and HPV L1 seropositive control women in the nested case-control study.

Risk factor	Among all cases and HPV L1 seropositive control women			
	CIN3/CIS		ICC	
	Controls / Cases	OR (95% CI)	Controls / Cases	OR (95% CI)
**Number of FTP** ^1^				
Never	64 / 51	1.0 (ref)	16 / 30	1.0 (ref)
Ever	250 / 288	**2.0 (1.2–3.2)**	122 / 131	0.6 (0.3–1.4)
1	71 / 71	1.4 (0.8–2.6)	21 / 26	0.9 (0.3–2.6)
2	99 / 132	**2.3 (1.3–4.0)**	69 / 53	**0.4 (0.2–1.0)**
3	47 / 46	**2.0 (1.0–4.0)**	24 / 28	0.7 (0.3–2.0)
≥4	24 / 28	**2.6 (1.2–5.9)**	7 / 16	1.2 (0.3–4.6)
*p for trend among ever ftp*		*0*.*2*		*0*.*3*
**Number of induced abortions** ^1^^**,**^^3^				
Never	127 / 166	1.0 (ref)	77 / 70	1.0 (ref)
Ever	58 / 45	**0.5 (0.3–0.8)**	29 / 41	1.7 (0.8–3.9)
1	42 / 36	**0.5 (0.3–0.9)**	18 / 26	2.0 (0.8–5.0)
≥2	16 / 9	**0.4 (0.1–0.9)**	11 / 15	1.4 (0.5–4.3)
**IUD use** ^2^^**,**^^3^				
Never	143 / 160	1.0 (ref)	78 / 106	1.0 (ref)
Ever	86 / 82	0.7 (0.4–1.1)	37 / 28	0.6 (0.3–1.2)
**OC use** ^2^				
Never	103 / 99	1.0 (ref)	59 / 56	1.0 (ref)
Ever	214 / 244	1.1 (0.7–1.6)	80 / 109	1.7 (0.9–3.3)
Past	182 / 197	1.1 (0.7–1.6)	70 / 86	1.6 (0.8–3.1)
Current	26 / 41	1.6 (0.8–3.2)	10 / 17	2.7 (0.8–9.1)
**Duration of OC use (years)** ^2^				
Never	103 / 99	1.0 (ref)	59 / 56	1.0 (ref)
≤4	82 / 82	1.0 (0.7–1.7)	32 / 36	1.4 (0.6–3.0)
5–9	53 / 54	0.9 (0.5–1.6)	15 / 22	2.1 (0.8–5.5)
≥10	60 / 94	1.6 (0.9–2.6)	31 / 42	1.8 (0.8–4.2)
*p for trend among OC users*		*0*.*1*		*0*.*3*
**HT use** ^4^^**,**^^5^				
Never	84 / 83	1.0 (ref)	54 / 67	1.0 (ref)
Ever	77 / 84	1.0 (0.6–1.7)	39 / 18	**0.3 (0.1–0.6)**
Past	23 / 17	0.6 (0.3–1.4)	10 / 8	0.5 (0.1–1.7)
Current	51 / 61	1.2 (0.7–2.2)	29 / 10	**0.2 (0.1–0.5)**
**Duration of HT use (years)** ^4^^**,**^^5^				
Never	84 / 83	1.0 (ref)	54 / 67	1.0 (ref)
≤1	18 / 28	1.4 (0.6–3.0)	21 / 11	**0.3 (0.1–0.8)**
2–4	24 / 15	0.5 (0.2–1.2)		
≥5	28 / 19	0.7 (0.3–1.6)	14 / 5	**0.2 (0.05–0.8)**
*p for trend among menopausal hormones users*		*0*.*3*		*-*

CIN3: cervical intraepithelial neoplasia grade 3; CIS: carcinoma in situ; ICC: invasive cervical cancer; OR: odds ratio; CI: confidence interval; FTP: full-term pregnancy; IUD: intrauterine device; OC: oral contraceptive; HT: hormone therapy. The number of cases and controls does not add up the total number because of missing values. Bold font indicates a statistically significant effect (p<0.05).

^1^ Unconditional regression models were adjusted by age, country, *Chlamydia Trachomatis* serology, *Human herpesvirus 2* serology, body mass index, marital status, level education, physical activity, smoking habits, OC use and duration and menopausal status with HT use.

^2^ Unconditional regression models were adjusted by age, country, *Chlamydia Trachomatis* serology, *Human herpesvirus 2* serology, body mass index, marital status, level education, physical activity, smoking habits, number of full-term pregnancies and menopausal status with HT use.

^3^ Excludes Bilthoven, Sweden and Norway because information was not collected for this variable.

^4^ Unconditional regression models were adjusted by age, country, *Chlamydia Trachomatis* serology, *Human herpesvirus 2* serology, body mass index, marital status, level education, physical activity, smoking habits, OC use and duration and number of full-term pregnancies.

^5^ Among post and perimenopausal women (excluding premenopausal women).

## Discussion

The results of this large prospective cohort study show that certain endogenous and exogenous hormonal factors appear to be related to cervical carcinogenesis. Thus the risk of cervical pre-cancer increased with increasing number of FTP and duration of OC use, and decreased with increasing years since last OC use among past users. For ICC, the risk increased with number of abortions and duration of OC use, and decreased with increasing duration of HT. A reduced risk of ICC was also observed among IUD users in the nested case-control study. Globally the associations were somewhat stronger in the cohort study than in the nested case-control study. The case-control study is useful to support the results obtained in the cohort study since it allowed for the additional adjustment of serological markers of HPV exposure and other STIs.

### OC use

Consistent with results from previously published pooled analyses [7,17], our study highlighted strong and positive associations between OC use and risk of cervical cancer and pre-cancer; specifically, the risk increased with duration of use and decreased with cessation of use. While the IARC collaborative study found a relative risk of 1.6 for long-term OC users with a significant trend, Moreno *et al*. observed a stronger association (OR = 4.0). In line with our findings, these two studies also found a reduced risk of cervical cancer for users who ceased OCs (RR = 0.8 and OR = 0.5, respectively). Other prospective studies did not find associations between OCs and CIN3/CC risk [18–20]. Analyses combining duration and recency of use evaluated in our study found a trend of lower CIN3/CIS risk with cessation of use for both short and long-term OC users, reinforcing the null association among past users and the higher risk among current users. The IARC collaborative study has also analyzed these factors in combination, obtaining similar patterns of trend, but unlike our study, for both CIN3/CIS and ICC risk.

Since HPV is the necessary cause of CC, analyses including some measure of HPV infection are needed to assess potential residual confounding. In our nested case-control study we included the adjustment by HPV serology, and we evaluated OC use and CC risk among all cases and HPV seropositive controls. As already discussed, these results were broadly similar to those obtained in the cohort study, suggesting that we can reasonably rule out a large confounding effect due to HPV infection in our associations. In the last 10 years, most of the published studies have also considered HPV infection in their analyses, and the results were globally comparable to those for all women. On the other hand, the very few studies that have used serology as HPV measurement found contradictory results [21–23]. Even though our study did not explicitly collect data related to sexual behavior we were able to adjust for serological markers of HPV, CT and HHV-2, as well as for marital status, which can all be considered good surrogate indicators of sexual behavior and risk of HPV exposure [24]. Since the results were very consistent with these adjustments, we were reassured that the potential effects of confounding by sexual behavior on our reported associations were minimal. Behavioral factors related to cervical cancer screening may also confound these associations. Again, even though the study did not collect individual data on screening practices, we systematically adjusted for surrogate markers of screening-related behavior such as number of pregnancies and variables related to contraceptive methods used in the past that may somewhat reduce the potential confounding effects due to screening practices. Also it is important to note that other studies that have adjusted for cervical screening did find positive associations with CC risk [7,17].

A possible mechanism to explain the associations between OC use and CC risk is that estrogens and progestogens may interact with hormone receptors, mainly progesterone, present in cervical tissue and influence the natural history of HPV infection. Specifically, sex steroid hormones are thought to enhance the expression of HPV 16 E6 and E7 oncogenes stimulating the degradation of p53 tumor suppressor genes and enhancing the ability of the viral DNA to transform cells and induce carcinogenesis [7,9,25,26]. These potential mechanisms are somewhat consistent with data from transgenic mouse models showing that estrogen and its nuclear receptor promote CC in combination with HPV oncogenes, but there are not in line with the observation that progesterone inhibits cervical carcinogenesis in mice [27,28].

### Parity

We found contrasting results between parity and risk of CIN3/CIS and ICC. Thus, while women with high parity had a higher risk of CIN3/CIS than nulliparous women, no associations were found with ICC. We also found that this association with high parity was present in each level of OC duration with a synergistic effect between the two variables. These results are in concordance with results from some [20,23] but not all [8,18,19,29] studies. The IARC collaborative study [8] and the IARC multicenter study [29] consistently found a significantly higher risk of CIN3/CIS and ICC among women with high parity. In contrast, two prospective studies conducted by Castle *et al*. [18,19] and a case-control study done in the US [23] did not find a significant association between parity and risk of CIN3/ICC. The lack of association between high parity and ICC risk found in our study could be explained by screening practices related to parity since it is likely that nulliparous women tend to be less screened than parous women. Hence, screening may act as a negative confounder, reducing the association between FTP and ICC risk. Globally the literature supports for an association between high parity and cervical cancer and pre-cancer risk.

As discussed in relation to OC use, these associations may be confounded by a number of other factors. However, adjustment by proxy measures of HPV exposure and sexual behavior did not change our risk estimates. In addition, two pooled analyses that adjusted their models by cervical screening practices obtained also similar results [8,29].

A possible biological mechanism for these associations could be that the elevated levels of estrogen and especially progesterone during pregnancy are responsible for the alterations in the squamo-columnal junction occurring during pregnancy, maintaining the transformation zone on the exocervix for many years. This would facilitate the direct exposure to HPV contributing to HPV persistence and progression to cervical neoplasia and cancer [8,20,29]. Another possible mechanism is immunosuppression linked to pregnancy which might enhance the role of HPV in cervical carcinogenesis [8].

### HT use

Our findings provide evidence for a reduction in ICC risk among peri- and postmenopausal women using HT, an effect that was stronger with longer duration of use. We found very few studies evaluating HT and CC [26,30]. Overall, the literature rules out positive associations. If anything, most studies found weak inverse associations that rarely reached statistical significance. In assessing HT, however, it is important to consider the hormonal composition of these treatments. The IARC review reported an increased risk for breast and endometrial cancer in the 1970s among postmenopausal women using estrogen only therapy, and this triggered health authorities to modify the hormonal composition of HT [6]. Even though our data on HT formulation were very limited, menopausal estrogens alone were associated with an increased risk of CIN3/CIS and combined HT were inversely associated with ICC. Other studies found some evidence for a reduced risk of ICC with ever use of estrogen therapy as well as duration as compared with never use [30–32]. When HT also included progesterone, the inverse association with ICC risk still remained both in our study as well as in other studies [30,33].

As noted previously, one of the limitations of this study is the lack of accurate individual screening history that may indeed influence the effects of HT use on CC risk. Thus, the inverse associations found with ICC, an outcome more susceptible to screening bias, could be somewhat overestimated. One possible explanation of this effect is that women who take HT are more frequently screened than non users, being consequently diagnosed and treated earlier of pre-cancerous lesions. Nevertheless, when we stratified analyses by proxy measures of screening such as parity and OC use, the inverse association remained (data not shown). The intrinsic mechanisms that might explain the biology of these potential associations are currently unknown. Data from HPV transgenic mouse models suggest that estrogens promote cervical carcinogenesis and progesterone inhibits CC [27,28]. However, our results, limited by the low number of subjects, are only partially consistent with these findings.

### Induced abortions

An increased risk of ICC and a reduced risk of CIN3/CIS were found among women reporting an induced abortion. Globally, the literature with regard to cervical cancer and pre-cancer risk and induced abortions is inconclusive and our results were in line with some [24,34–36] but not all [29,37,38] studies. More data are needed to better explore these potential associations.

### Menstrual lifespan

We observed a decreased risk of both CIN3/CIS and ICC risk with increasing cumulative years of menstrual cycles. Longer menstrual lifespan is implicitly associated with shorter OC use duration and lower parity. However, the advantage of using menstrual lifespan is that includes in a single indicator combined information on the cumulative exposure to some endogenous and exogenous hormones taking into account the actual hormonal lifespan of the woman, providing thus more robustness in the risk estimates. It is interesting to note that even though this variable has been used in studies of other hormone-related cancers such as breast, ovarian and endometrial, no previous studies of CC have been identified in the literature.

### IUD use

We found a non significant decreased risk of both CIN3/CIS and ICC among IUD users in the nested case-control study that reached statistical significance in the analyses restricted to all cases and HPV seropositive controls when combining CIN3/CIS and ICC cases (OR = 0.7, 95% CI: 0.5–0.96; data not shown) and in the analyses restricted to HPV seropositive women for ICC risk (OR = 0.3, 95% CI: 0.1–0.9; data not shown). The full case-control study and the different stratified analyses consistently found that IUD use was inversely associated with both CIN3/CIS and ICC risk. Few studies have evaluated associations between IUD use and CC risk with inconsistent results [20,23,37,39]. In a population-based study conducted in the US, Shields and colleagues found a protective effect between duration of IUD and ICC (OR = 0.3, 95% CI: 0.2–0.8, for ≥5 years of IUD use) [23]. In a large pooled analysis, Castellsagué and colleagues reported a strong inverse association between IUD use and ICC (OR = 0.5, 95% CI: 0.4–0.7) [39]. Other studies, however, have not confirmed these findings, although the use of a combined outcome (CIN3/CIS and ICC) or a low number of cases could explain these discrepancies [20,37]. Even though we adjusted for major risk factors such as past exposure to HPV, STIs and sexual behavior, it is true that we cannot completely rule out a residual confounding effect exerted by CC screening. It is important to note that the study by Castellsagué did adjust the analyses for cervical HPV DNA, age at first sex, and number of PAP smears, and still found a statistically significant inverse association. One possible mechanism that could explain this potential protective effect is through a device-related inflammatory reaction in the cervix and endocervix that could influence the subsequent likelihood of HPV persistence and/or progression to CC [39].

### SCC versus adenocarcinoma

Globally, the associations found between both exogenous and endogenous hormones and SCC risk were comparable to those obtained in models evaluating CIN3/CIS and ICC risk (data not shown). Concerning the risk of invasive adenocarcinoma, even though there were very few cases, an association was found with increasing number of FTP, in addition to OC use and HT, in the cohort study (data not shown). In concordance with our study, the IARC collaborative reanalysis evaluating risk factors for SCC and adenocarcinoma obtained an increased risk for each histological type with increasing duration of OC use [40]. Contrary to this and to another study [41], our results did not indicate that high parity is a risk cofactor for developing SCC, probably due to screening bias. Concerning HT use and contrary to our findings, Lacey and colleagues found that exogenous estrogens, especially unopposed estrogens, were positively associated with adenocarcinomas [30]. However, since both studies accounted for small number of cases, these findings should be cautiously interpreted.

### Strengths and limitations

Our study has several strengths including its prospective, population-based design in most of the countries, a large sample size that embraces various regions in Europe, and the inclusion of two disease outcomes: CIN3/CIS and ICC. In addition, our inclusion of a nested case-control study within the cohort allowed us to determine and take into account biomarkers of past exposure to STIs such as HPV, CT and HHV-2.

Nevertheless, our study design failed to consider direct markers of HPV infection, cervical cancer screening practices or sexual behavior, not only at recruitment but also during follow-up, as none of the assessed risk factors or potential confounding variables was reassessed after recruitment. Since these risk factors could and most likely did change in an unknown direction during follow-up, our associations may have led to under- or overestimation of the real effects on CC risk. Another limitation was the low sensitivity of the HPV serology technique, as only half of infected women seroconvert, and a potential misclassification cannot be totally ruled out [12].

### Conclusions

This study contributes strong evidence to consider high parity and long-term OC use important risk factors for cervical cancer and pre-cancer. These risk factors would act not independently but rather as cofactors that interact with HPV to induce cervical carcinogenesis. Our study also provides some evidence that IUD use and HT confer a reduced risk of cervical cancer, but these findings need further confirmation. Despite the clear involvement of hormones in cervical carcinogenesis, and from a public health point of view, adherence to current screening recommendations will certainly minimize the potential increased risk of CC associated with some of these hormonal risk factors.

## Supporting Information

S1 Table Baseline characteristics of cases and non-cases in the cohort study. (DOCX)

S2 Table Risk of CIN3/CIS and ICC of the cervix according to the combined effect of number of full-term pregnancies and duration of use of hormonal contraceptives. (DOCX)

S1 Text STROBE checklist. (DOC)

## Abbreviations

CC: Cervical Cancer
CI: Confidence Interval
CIN3: Cervical Intraepithelial Neoplasia grade 3
CIS: Carcinoma In Situ
CT: Chlamydia trachomatis
EPIC: European Prospective Investigation into Cancer and Nutrition
FTP: Full-term pregnancy
HHV-2: Human herpesvirus 2
HPV: Human Papillomavirus
HR: Hazard Ratio
HT: Hormone Therapy
IARC: International Agency for Research on Cancer
ICC: Invasive Cervical Cancer
IUD: Intrauterine Device
OR: Odds Ratio
OC: Oral Contraceptives
SCC: Squamous Cell Carcinoma
STIs: Sexually Transmitted Infections
