# Correction: The Influence of Hormonal Factors on the Risk of Developing Cervical Cancer and Pre-Cancer: Results from the EPIC Cohort

**DOI:** 10.1371/journal.pone.0151427

**Published:** 2016-03-08

**Authors:** Esther Roura, Noémie Travier, Tim Waterboer, Silvia de Sanjosé, F. Xavier Bosch, Michael Pawlita, Valeria Pala, Elisabete Weiderpass, Núria Margall, Joakim Dillner, Inger T. Gram, Anne Tjønneland, Christian Munk, Domenico Palli, Kay-Tee Khaw, Kim Overvad, Françoise Clavel-Chapelon, Sylvie Mesrine, Agnès Fournier, Renée T. Fortner, Jennifer Ose, Annika Steffen, Antonia Trichopoulou, Pagona Lagiou, Philippos Orfanos, Giovanna Masala, Rosario Tumino, Carlotta Sacerdote, Silvia Polidoro, Amalia Mattiello, Eiliv Lund, Petra H. Peeters, H. B(as). Bueno-de-Mesquita, J. Ramón Quirós, María-José Sánchez, Carmen Navarro, Aurelio Barricarte, Nerea Larrañaga, Johanna Ekström, David Lindquist, Annika Idahl, Ruth C. Travis, Melissa A. Merritt, Marc J. Gunter, Sabina Rinaldi, Massimo Tommasino, Silvia Franceschi, Elio Riboli, Xavier Castellsagué

There are errors in the Funding section. The complete, correct Funding section is as follows:

The work was partially supported by grants from the Instituto de Salud Carlos III (Spanish Government) (grants FIS PI08/1308, FIS PI11/02096, PI13/00053, RCESP C03/09, RTICESP C03/10, RTIC RD06/0020/0095, RD12/0036/0056, RD12/0036/0018, and CIBERESP) and from the Agència de Gestió d'Ajuts Universitaris i de Recerca—Generalitat de Catalunya (Catalonian Government) (grants AGAUR 2005SGR00695, 2009SGR939 and 2009SGR126, 2014SGR1077, 2014SGR756, 2014SGR2016), European Regional Development fund (FEDER) "A way to build Europe". The coordination of EPIC is financially supported by the European Commission (DG-SANCO) and the International Agency for Research on Cancer. The national cohorts are supported by the Health Research Fund (FIS) of the Spanish Ministry of Health (Exp P10710130), Regional Governments of Andalucía, Asturias, Basque Country, Murcia (no. 6236), Navarra and the Catalan Institute of Oncology, La Caixa (BM 06–130), RTICC-RD06/10091 (Spain); Danish Cancer Society (Denmark); Ligue contre le Cancer, Institut Gustave Roussy, Mutuelle Générale de l'Education Nationale, Institut National de la Santé et de la Recherche Médicale (INSERM) (France); Deutsche Krebshilfe, Deutsches Krebsforschungszentrum and Federal Ministry of Education and Research (Germany); the Hellenic Health Foundation (Greece); Italian Association for Research on Cancer (AIRC) and National Research Council (Italy); Dutch Ministry of Public Health, Welfare and Sports (VWS), Netherlands Cancer Registry (NKR), LK Research Funds, Dutch Prevention Funds, Dutch ZON (Zorg Onderzoek Nederland), World Cancer Research Fund (WCRF) and Statistics Netherlands (The Netherlands); Swedish Cancer Society, Swedish Scientific Council and Regional Government of Skåne and Västerbotten (Sweden); Cancer Research UK, Medical Research Council (United Kingdom); Norwegian Research Council, Norwegian Cancer Society, University of Tromso (Norway). The funders had no role in study design, data collection and analysis, decision to publish, or preparation of the manuscript.
