# Supplementary material for: The Influence of Hormonal Factors on the Risk of Developing Cervical Cancer and Pre-Cancer: Results from the EPIC Cohort
Source: PLoS One. 2016 Jan 25;11(1):e0147029. doi: 10.1371/journal.pone.0147029 (PMC4726518; doi:10.1371/journal.pone.0147029)
Supplement: S1 Table — (DOCX) [file pone.0147029.s001.docx]

**S1 Table. Baseline characteristics of cases and non-cases in the cohort study**

| **Characteristics of subjects in the cohort study** | **Person-years** | **Non-cases**  **(N=306,971)** | **Cases** | |
| --- | --- | --- | --- | --- |
|  |  |  | **CIN3/CIS**  **(N=804)** | **ICC**  **(N=261)** |
|  |  | **N (%)** | **N (%)** | **N (%)** |
| **Country** |  |  |  |  |
| France | 710,002 | 62,479 (20.4) | 51 (6.3) | 30 (11.5) |
| Italy | 244,437 | 28,279 (9.2) | 22 (2.7) | 18 (6.9) |
| Spain | 229,273 | 23,190 (7.6) | 22 (2.7) | 23 (8.8) |
| United Kingdom | 420,545 | 48,975 (16.0) | 298 (37.1) | 35 (13.4) |
| The Netherlands | 197,604 | 22,427 (7.3) | 3 (0.4) | 19 (7.3) |
| Greece | 102,361 | 14,031 (4.6) | 4 (0.5) | 13 (5.0) |
| Germany | 201,023 | 24,148 (7.9) | 52 (6.5) | 25 (9.6) |
| Sweden | 285,082 | 26,709 (8.7) | 167 (20.8) | 44 (16.9) |
| Denmark | 192,795 | 24,893 (8.1) | 64 (8.0) | 33 (12.6) |
| Norway | 192,114 | 31,840 (10.4) | 112 (15.0) | 21 (8.0) |
| **Age at recruitment (years)** |  |  |  |  |
| <30 | 87,347 | 10,216 (3.3) | 125 (15.5) | 9 (3.4) |
| 30-39 | 275,337 | 31,383 (10.2) | 180 (22.4) | 32 (12.3) |
| 40-49 | 942,999 | 103,483 (33.7) | 275 (34.2) | 95 (36.4) |
| 50-59 | 989,738 | 109,403 (35.6) | 186 (23.1) | 85 (32.6) |
| ≥60 | 479,814 | 52,486 (17.1) | 38 (4.7) | 40 (15.3) |
| Mean age (5th - 95th percentile) | - | 50.3 (32.5-65.8) | 42.4 (24.4-59.8) | 49.3 (31.4-64.8) |
| **Marital status** |  |  |  |  |
| Single | 1,780,208 | 28,807 (11.6) | 141 (21.0) | 27 (13.8) |
| Married/living together | 275,572 | 195,074 (78.3) | 426 (63.6) | 133 (68.2) |
| Divorced/separated | 131,296 | 14,674 (5.9) | 93 (13.9) | 31 (15.9) |
| Widowed | 90,828 | 10,559 (4.2) | 10 (1.5) | 4 (2.1) |
| Missing † | 497,331 | 57,857 | 134 | 66 |
| **Smoking status** |  |  |  |  |
| Never smokers | 1,581,045 | 170,511 (57.0) | 325 (41.0) | 108 (41.7) |
| Past smokers | 608,927 | 68,524 (22.9) | 198 (25.0) | 66 (25.5) |
| Current smokers | 516,427 | 60,278 (20.1) | 270 (34.0) | 85 (32.8) |
| Missing † | 68,837 | 7,658 | 11 | 2 |
| **Number of FTP** |  |  |  |  |
| 0 | 397,014 | 45,228 (16.0) | 191 (27.7) | 45 (19.9) |
| 1 | 399,396 | 44,007 (15.6) | 116 (16.8) | 36 (15.9) |
| 2 | 1,032,936 | 114,956 (10.8) | 237 (34.4) | 82 (36.3) |
| 3 | 485,631 | 54,107 (19.2) | 94 (13.6) | 40 (17.7) |
| ≥4 | 212,074 | 23,545 (8.4) | 51 (7.4) | 23 (10.2) |
| Missing † | 248,184 | 25,128 | 115 | 35 |
| **OC use** |  |  |  |  |
| Never users | 1,090,625 | 121,117 (41.6) | 169 (23.9) | 76 (32.8) |
| Past users | 1,366,500 | 152,658 (52.4) | 411 (58.1) | 134 (57.8) |
| Current users | 150,646 | 17,384 (6.0) | 127 (18.0) | 22 (9.5) |
| Missing † | 167,464 | 15,812 | 97 | 29 |
| **Menopausal status** |  |  |  |  |
| Premenopausal | 1,044,990 | 116,583 (38.0) | 490 (60.9) | 117 (44.8) |
| Perimenopausal | 480,394 | 53,095 (17.3) | 123 (15.3) | 42 (16.1) |
| Postmenopausal | 1,249,851 | 137,293 (44.7) | 191 (23.8) | 102 (39.1) |

CIN3: cervical intraepithelial neoplasia grade 3; CIS: carcinoma in situ; ICC: invasive cervical cancer; FTP: full-term pregnancy; OC: oral contraceptive.

† Not included in the percentage distribution of the variable.
