# Supplementary material for: The Influence of Hormonal Factors on the Risk of Developing Cervical Cancer and Pre-Cancer: Results from the EPIC Cohort
Source: PLoS One. 2016 Jan 25;11(1):e0147029. doi: 10.1371/journal.pone.0147029 (PMC4726518; doi:10.1371/journal.pone.0147029)
Supplement: S2 Table — (DOCX) [file pone.0147029.s002.docx]

**S2 Table. Risk of CIN3/CIS and ICC of the cervix according to the combined effect of number of full-term pregnancies and duration of use of hormonal contraceptives**

| **Risk factor** | **Cohort study** | | | | **Nested case-control study** | | | |
| --- | --- | --- | --- | --- | --- | --- | --- | --- |
|  | **CIN3/CIS** | | **ICC** | | **CIN3/CIS** | | **ICC** | |
|  | **Non-cases /**  **Cases** | **HR (95% CI) ^1^** | **Non-cases /**  **Cases** | **HR (95% CI) ^1^** | **Controls /**  **Cases** | **OR (95% CI) ^2^** | **Controls /**  **Cases** | **OR (95% CI) ^2^** |
| **Never OC use** |  |  |  |  |  |  |  |  |
| Nulliparous | 18,786 / 21 | 1.0 (ref) | 18,807 / 14 | 1.0 (ref) | 46 / 9 | 1.0 (ref)) | 19 / 11 | 1.0 (ref) |
| 1 FTP | 17,414 / 22 | **2.1 (1.1-3.8)** | 17,436 / 11 | 0.9 (0.4-1.9) | 46 / 14 | 2.2 (0.8-6.5) | 16 / 9 | 1.1 (0.3-4.6) |
| 2-3 FTP | 67,745 / 97 | **2.9 (1.7-4.7)** | 67,842 / 39 | 0.8 (0.4-1.4) | 100 / 54 | **4.7 (1.8-12.4)** | 90 / 26 | 0.5 (0.2-1.5) |
| ≥4 FTP | 12,040 / 18 | **4.0 (2.1-7.7)** | 12,058 / 9 | 1.0 (0.4-2.3) | 18 / 13 | **6.0 (1.8-21.4)** | 11 / 7 | 2.1 (0.5-8.4) |
| **OC use < 5 years** |  |  |  |  |  |  |  |  |
| Nulliparous | 10,132 / 64 | **2.3 (1.4-3.8)** | 10,196 / 9 | 1.4 (0.6-3.4) | 38 / 16 | 1.8 (0.6-4.9) | 11 / 6 | 1.1 (0.2-5.4) |
| 1 FTP | 10,022 / 26 | **2.1 (1.2-3.8)** | 10,048 / 5 | 0.7 (0.3-2.1) | 31 / 15 | **3.3 (1.1-9.9)** | 8 / 4 | 0.6 (0.1-3.6) |
| 2-3 FTP | 43,114 / 96 | **2.5 (1.5-4.0)** | 43,210 / 31 | 1.1 (0.6-2.1) | 96 / 44 | **3.6 (1.4-9.5)** | 50 / 20 | 0.9 (0.3-2.6) |
| ≥4 FTP | 5,560 / 17 | **4.4 (2.3-8.5)** | 5,577 / 5 | 1.1 (0.4-3.3) | 13 / 7 | **4.5 (1.2-16.4)** | 3 / 4 | 2.4 (0.3-17.7) |
| **OC use** ≥ **5 years** |  |  |  |  |  |  |  |  |
| Nulliparous | 14,056 / 94 | **2.5 (1.6-4.1)** | 14,150 / 18 | 1.9 (0.9-4.0) | 46 / 24 | **3.6 (1.3-10.1)** | 10 / 11 | 1.6 (0.4-6.8) |
| 1 FTP | 14,458 / 65 | **3.2 (1.9-5.4)** | 14,523 / 19 | 1.7 (0.8-3.5) | 53 / 42 | **4.5 (1.8-13.5)** | 17 / 12 | 1.3 (0.4-4.8) |
| 2-3 FTP | 50,117 / 125 | **2.6 (1.6-4.2)** | 50,242 / 47 | 1.3 (0.7-2.5) | 134 / 73 | **4.2 (1.7-10.6)** | 67 / 34 | 1.1 (0.4-3.4) |
| ≥4 FTP | 4,863 / 15 | **4.4 (2.2-8.6)** | 4,878 / 9 | **2.4 (1.0-5.8)** | 10 / 8 | **6.5 (1.7-24.8)** | 3 / 5 | 1.3 (0.1-12.7) |
| P value ^3^ | **0.004** | | 0.9 | | 0.2 | | 0.8 | |

CIN3: cervical intraepithelial neoplasia grade 3; CIS: carcinoma in situ; ICC: invasive cervical cancer; HR: hazard ratio; OR: odds ratio; CI: confidence interval; FTP: full-term pregnancy; OC: oral contraceptives; HT: hormone therapy.

^1^ Models were adjusted by body mass index, marital status, level education, physical activity, smoking habits and menopausal status with HT use. ^2^ Conditional regression models were adjusted by HPV L1 serology, *Chlamydia trachomatis* serology, *Human herpesvirus 2* serology, body mass index, marital status, level education, physical activity, smoking habits and menopausal status with HT use. See methods for list matching variables. ^3^ P value for interaction between number of FTP and duration of OC use.

The number of cases does not add up the total number of cases because of missing values.

Bold font signifies a statistically significant effect (p<0.05).
