# Supplementary material for: The Influence of Hormonal Factors on the Risk of Developing Cervical Cancer and Pre-Cancer: Results from the EPIC Cohort
Source: PLoS One. 2016 Jan 25;11(1):e0147029. doi: 10.1371/journal.pone.0147029 (PMC4726518; doi:10.1371/journal.pone.0147029)
Supplement: S1 Text — (DOC) [file pone.0147029.s003.doc]

**S1 - STROBE Statement**—checklist of items that should be included in reports of observational studies

|  | | Item No | Recommendation |
| --- | --- | --- | --- |
| **Title and abstract** | | 1 | (*a*) Indicate the study’s design with a commonly used term in the title or the abstract **– ABSTRACT** |
|  |  |  | (*b*) Provide in the abstract an informative and balanced summary of what was done and what was found **– ABSTRACT** |
| Introduction | | | |
| Background/rationale | | 2 | Explain the scientific background and rationale for the investigation being reported **– INTRODUCTION** |
| Objectives | | 3 | State specific objectives, including any prespecified hypotheses **– INTRODUCTION** |
| Methods | | | |
| Study design | | 4 | Present key elements of study design early in the paper **– METHODS (The EPIC cohort study)** |
| Setting | | 5 | Describe the setting, locations, and relevant dates, including periods of recruitment, exposure, follow-up, and data collection **– METHODS (The EPIC cohort study, Identification of cases and follow-up)** |
| Participants | | 6 | (*a*) *Cohort study*—Give the eligibility criteria, and the sources and methods of selection of participants. Describe methods of follow-up **– METHODS (Study population, Identification of cases and follow-up)**  *Case-control study*—Give the eligibility criteria, and the sources and methods of case ascertainment and control selection. Give the rationale for the choice of cases and controls **– METHODS (Study population, Identification of cases and follow-up, Nested case-control study)**  *Cross-sectional study*—Give the eligibility criteria, and the sources and methods of selection of participants **– NOT APPLICABLE** |
|  |  |  | (*b*) *Cohort study*—For matched studies, give matching criteria and number of exposed and unexposed **– NOT APPLICABLE**  *Case-control study*—For matched studies, give matching criteria and the number of controls per case **– METHODS (Nested case-control study)** |
| Variables | | 7 | Clearly define all outcomes, exposures, predictors, potential confounders, and effect modifiers. **– METHODS (Identification of cases and follow-up, Nested case-control study , Serological testing, Statistical analyses)**  Give diagnostic criteria, if applicable **– METHODS (Identification of cases and follow-up)** |
| Data sources/ measurement | | 8* | For each variable of interest, give sources of data and details of methods of assessment (measurement). **– METHODS (Identification of cases and follow-up, Nested case-control study , Serological testing, Statistical analyses)**  Describe comparability of assessment methods if there is more than one group **– NOT APPLICABLE** |
| Bias | | 9 | Describe any efforts to address potential sources of bias **– METHODS (Statistical analyses), DISCUSSION** |
| Study size | | 10 | Explain how the study size was arrived at **– METHODS (Study population, Identification of cases and follow-up, Nested case-control study)** |
| Quantitative variables | | 11 | Explain how quantitative variables were handled in the analyses. If applicable, describe which groupings were chosen and why **– METHODS (Statistical analyses)** |
| Statistical methods | | 12 | (*a*) Describe all statistical methods, including those used to control for confounding **– METHODS (Statistical analyses)** |
|  |  |  | (*b*) Describe any methods used to examine subgroups and interactions **– METHODS (Statistical analyses)** |
|  |  |  | (*c*) Explain how missing data were addressed **– METHODS (Statistical analyses)** |
|  |  |  | (*d*) *Cohort study*—If applicable, explain how loss to follow-up was addressed **– METHODS (Identification of cases and follow-up)**  *Case-control study*—If applicable, explain how matching of cases and controls was addressed **– METHODS (Nested case-control study)**  *Cross-sectional study*—If applicable, describe analytical methods taking account of sampling strategy **– NOT APPLICABLE** |
|  |  |  | (*e*) Describe any sensitivity analyses **– RESULTS, DISCUSSION (for HT use, Induced abortions, IUD use)** |
| Results | | | |
| Participants | 13* | (a) Report numbers of individuals at each stage of study—eg numbers potentially eligible, examined for eligibility, confirmed eligible, included in the study, completing follow-up, and analysed **– METHODS (The EPIC cohort study, Study population, Identification of cases and follow-up, Nested case-control study)** | |
|  |  | (b) Give reasons for non-participation at each stage **– METHODS (Study population, Identification of cases and follow-up, Nested case-control study)** | |
|  |  | (c) Consider use of a flow diagram **– NOT APPLICABLE** | |
| Descriptive data | 14* | (a) Give characteristics of study participants (eg demographic, clinical, social) and information on exposures and potential confounders **– RESULTS (TABLE 1)** | |
|  |  | (b) Indicate number of participants with missing data for each variable of interest **– RESULTS (TABLE 1)** | |
|  |  | (c) *Cohort study*—Summarise follow-up time (eg, average and total amount) **– METHODS (Identification of cases and follow-up), RESULTS (TABLE 1)** | |
| Outcome data | 15* | *Cohort study*—Report numbers of outcome events or summary measures over time **–RESULTS (TABLE 1)** | |
|  |  | *Case-control study—*Report numbers in each exposure category, or summary measures of exposure **– RESULTS (TABLES 2 AND 3)** | |
|  |  | *Cross-sectional study—*Report numbers of outcome events or summary measures **– NOT APPLICABLE** | |
| Main results | 16 | (*a*) Give unadjusted estimates and, if applicable, confounder-adjusted estimates and their precision (eg, 95% confidence interval). Make clear which confounders were adjusted for and why they were included **– METHODS (Statistical analyses), RESULTS (TABLES 2 AND 3)** | |
|  |  | (*b*) Report category boundaries when continuous variables were categorized **– RESULTS (TABLES 1, 2, 3 AND 4)** | |
|  |  | (*c*) If relevant, consider translating estimates of relative risk into absolute risk for a meaningful time period **– NOT RELEVANT GIVEN THE CONTEXT** | |
| Other analyses | 17 | Report other analyses done—eg analyses of subgroups and interactions, and sensitivity analyses **– RESULTS (TABLE 4, S1 TABLE)** | |
| Discussion | | | |
| Key results | 18 | Summarise key results with reference to study objectives **– DISCUSSION (first paragraph)** | |
| Limitations | 19 | Discuss limitations of the study, taking into account sources of potential bias or imprecision. Discuss both direction and magnitude of any potential bias **– DISCUSSION (OC use, Parity, HT use, IUD use, Strengths and limitations)** | |
| Interpretation | 20 | Give a cautious overall interpretation of results considering objectives, limitations, multiplicity of analyses, results from similar studies, and other relevant evidence **– DISCUSSION (CONCLUSIONS)** | |
| Generalisability | 21 | Discuss the generalisability (external validity) of the study results **– DISCUSSION (Strengths and limitations** | |
| Other information | | | |
| Funding | 22 | Give the source of funding and the role of the funders for the present study and, if applicable, for the original study on which the present article is based **– FUNDING** | |

*Give information separately for cases and controls in case-control studies and, if applicable, for exposed and unexposed groups in cohort and cross-sectional studies.

**Note:** An Explanation and Elaboration article discusses each checklist item and gives methodological background and published examples of transparent reporting. The STROBE checklist is best used in conjunction with this article (freely available on the Web sites of PLoS Medicine at http://www.plosmedicine.org/, Annals of Internal Medicine at http://www.annals.org/, and Epidemiology at http://www.epidem.com/). Information on the STROBE Initiative is available at www.strobe-statement.org.
